# Synthesis of New Phosphorus-Containing (Co)Polyesters Using Solid-Liquid Phase Transfer Catalysis and Product Characterization 

**DOI:** 10.3390/molecules17089090

**Published:** 2012-07-31

**Authors:** Smaranda Iliescu, Maite-Gyl Augusti, Eugenia Fagadar-Cosma, Nicoleta Plesu, Gheorghe Fagadar-Cosma, Lavinia Macarie, Adriana Popa, Gheorghe Ilia

**Affiliations:** 1 Institute of Chemistry, Romanian Academy, 24 Mihai Viteazul Bvd., Timisoara 300-223, Romania; Email: smail@acad-icht.tm.edu.ro (S.I.); efagadar@yahoo.com (E.F.-C.); plesu_nicole@yahoo.com (N.P.); lavi_mac@yahoo.com (L.M.); apopa_ro@yahoo.com (A.P.); 2 Instituto Tecnológico de la Energía, Av. Juan de la Cierva, 24 Parque Tecnológico de Valencia, Paterna 46980 (Valencia), Spain; Email: mayte.gil@ite.es; 3 Polytechnic University of Timisoara T. Lalescu Street, No. 2, Timisoara 300-223, Romania; Email: gfagadar@yahoo.com

**Keywords:** phase transfer catalysis, solid polymer electrolytes, phosphorus containing (co)polyesters

## Abstract

This paper is directed towards the development of safe, and thermally stable solid polymer electrolytes. Linear phosphorus-containing (co)polyesters are described, including their synthesis, thermal analysis, conductivity, and non-flammability. Polycondensation of phenylphosphonic dichloride (PPD) with poly(ethylene glycol) (PEG 12000) with and without bisphenol A (BA) was carried out using solid-liquid phase transfer catalysis. Potassium phosphate is used as base. Yields in the range of 85.0–88.0%, and inherent viscosities in the range of 0.32–0.58 dL/g were obtained. The polymers were characterized by gel permeation chromatography, FT-IR, ^1^H- and ^31^P-NMR spectroscopy and thermal analysis. Their flammability was investigated by measuring limiting oxygen index values. The polymers are flame retardants and begin to lose weight in the 190 °C–231 °C range. Solid phosphorus- containing (co)polyesters were complexed with lithium triflate and the resulting ionic conductivity was determined. Conductivities in the range of 10^−7^–10^−8^ S cm^−1^ were obtained.

## 1. Introduction

**Scheme 1:**
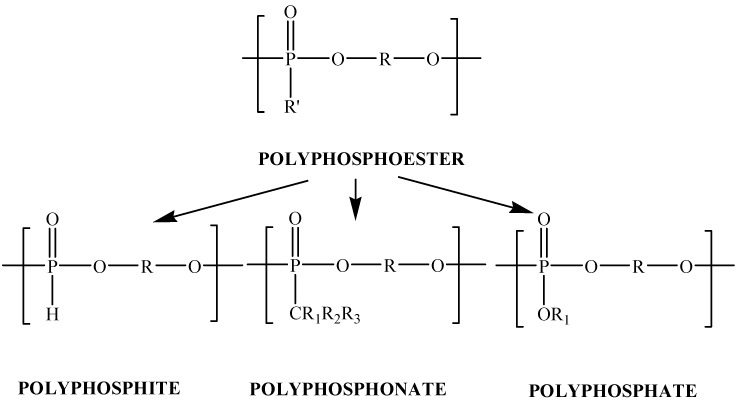
Class of polyphosphoesters.

Polyphosphoesters are an important class of phosphorus-containing polymers because of their specific properties, e.g., a good flame resistance, plasticity, lubricant properties and good heat stability. Polyphosphoesters contain repeated phosphoester bonds in the backbone and are structurally versatile, biocompatible, and biodegradable through hydrolysis as well as enzymatic digestion under physiological conditions. Depending on the nature of the side group connected to the phosphorus atom, the polymers are also called polyphosphates, polyphosphonates, or polyphosphites, as shown in Scheme 1 [1].

One of the present preoccupations lies in the intensification of the efforts concerning fire proofing of macromolecular compounds which although beneficial from a technical and economic point of view are in danger of ignition [2,3]. In the last decade, a remarkable interest was given to some categories of phosphorus-containing polymers, such as polyphosphates, essentially because they are used in the synthesis of poly(alkylenephosphate) biopolymers [4].

Recently, organophosphorus polymers have regained researchers’ interest as polymer electrolytes. Solid polymer electrolytes (SPE) are promising materials for electrochemical device applications, namely, high energy density rechargeable batteries, fuel cells, supercapacitors, electrochromic displays, *etc*. [5].

Solid polymer electrolyte systems with fire-retardant polymer matrixes have been investigated in only a few cases. Novel safe and non-flammable phosphorus-containing polymers, namely phosphorus-based electrolytes based on phosphate as a linking agent for poly(ethylene glycol) (P-PEG) were synthesized. Ionic conductivity and thermal behavior of (P-PEG) series–LiCF_3_SO_3_ complexes were investigated with various compositions, salt concentrations and temperatures [6,7,8,9].

Among the main ways of obtaining phosphorus-containing polymers, especially polyphosphonates and polyphosphates, respectively, polyaddition reactions [10,11], polymerization [12], ring opening polymerization [13], polytransesterification [14], and polycondensation [15,16,17,18], phase transfer catalysis (PTC) or interfacial polycondensation reactions [19,20,21] have seen increasing development in the last years.

The PTC method has attracted much interest due to the simplicity of the operations and technological devices required, economy of energy and raw materials and, in particular, as modern and new perspectives in modern organic synthesis. In our earlier papers we have reported the possibility of obtaining phosphorus-containing polymers, particulalry polyphosphonates and polyphosphates, by PTC in liquid-liquid system [22], gas-liquid system [23], solid-liquid [24], and inverse phase transfer catalysis [25].

Polyphosphoester electrolytes were synthesized by polycondensation reactions of phosphorus oxychloride or methyldiclorophosphate with PEG in order to improve the ionic conductivity at ambient temperature in further applications as SPE membranes [6,7,8,9]. This paper presents for the first time the synthesis of phosphorus-containing (co)polyester electrolytes by phase transfer catalysis. PTC (liquid-liquid or gas-liquid polycondensation) has drawbacks in obtaining polymers with high molar masses, because the presence of water can lead to side reactions such as hydrolysis of the phosphorus-chloride bond of the reagent or chain end-groups of the polymer [24]. The presence of water in the membranes for lithium rechargeable batteries leads to decrease of their electrochemical windows [26].

**Scheme 2:**
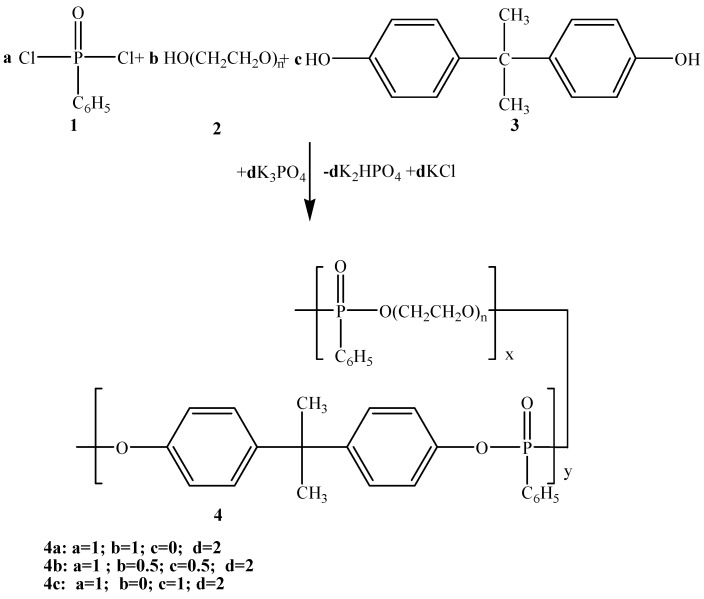
Preparation of random phosphorus containing (co)polyesters **4a–c**.

The conversion of liquid-liquid or gas-liquid PTC into solid-liquid PTC proves to be advantageous for suppression of the side reactions in order to obtain a polymer candidate for solid polymer electrolytes, without any impurity and moisture. This paper presents the synthesis of linear phosphorus-containing (co)polyesters by solid-liquid phase transfer catalysis polycondensation of phenylphosphonic dichloride (PPD) with poly(ethylene glycol) (PEG 12000) with and without bisphenol A (BA) (Scheme 2).

The thermal stability and flammability of the (co)polymers have been investigated. Ionic conductivity of (co)polymers **4a** and **4b** was also determined.

## 2. Results and Discussion

The aim of this work was to apply phase-transfer catalysis in a solid-liquid system for the first time in solid polymer electrolytes, as an eco-friendly and economical procedure for making phosphorus- containing polymers by using potassium phosphate as base.

Kim [6,7] and Morris [8] synthesized phosphorus solid polymer electrolytes by polycondensation in solution, when triethylamine was used as the scavenger of HCl side products. With this scavenging with a tertiary amine, a thick, non-stirrable slurry and low yields were obtained.

In order to prevent the degradation of (co)polymers, reduce the competing end reactions (the presence of water can lead to side reactions such as hydrolysis of the phosphorus-chloride bond of the reagent or of the chain end-groups of the polymer, the presence of water in the membrane leads to a decrease of electrochemical windows) and get data for preparing thermally stable (co)polymers we have synthesized phosphorus solid polymer electrolytes by PTC in a solid-liquid system, where potassium phosphate is used as base [24].

Phosphorus-containing (co)polymers **4a–c** were synthesized by solid-liquid PTC polycondensation of phenylphosphonic dichloride with poly(ethylene glycol) 12,000 with and without bisphenol A. Copolyphosphonate **4b** is a new product. This method requires no product purification and affords the desired compounds in reasonable yield without the formation of unwanted side products.

The potassium phosphate acts as acid scavenger without water formation according to the reaction HCl + K_3_PO_4_ → KCl + K_2_HPO_4_ [27]. Table 1 shows the yields, inherent viscosities, molecular weights and phosphorus content of **4a–c** obtained by PTC polycondensation in solid–liquid technique.

**Table 1:** Results of solid–liquid PTC polycondensation of PPD with PEG and/or BA ^a^.

(Co)polym	η, %	η_inh_, ^b^ dL/g	Mn × 10^−4^	Mw × 10^−4^	polydisp.	P (%) ^c^	
						calc	exp
**4a**	85.4	0.58	2.63	3.0	1.14	0.25	0.22
**4b**	86.5	0.55	2.30	2.77	1.16	0.25	0.20
**4c**	88.0	0.32	0.54	0.62	1.15	8.85	8.20

^a^ reaction conditions: 0.005 mol PEG 12000 and/or BA, 0.01 mol K_3_PO_4_, 20 mL (2-MeTHF), 0.005 mol PPD and 10 mL 2-MeTHF, 2 h, 600 rpm, 45 °C; ^b^ measured at a concentration of 0.5 g/dL in tetrachloroethane, at 30 °C; ^c^ determined by Schöniger method.

**Figure 1:**
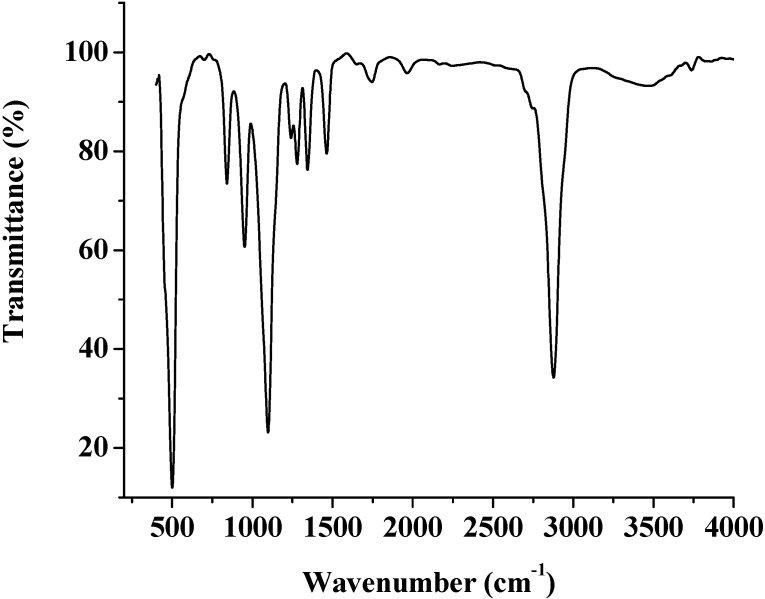
IR spectrum of polyphosphonate **4b**.

**Figure 2:**
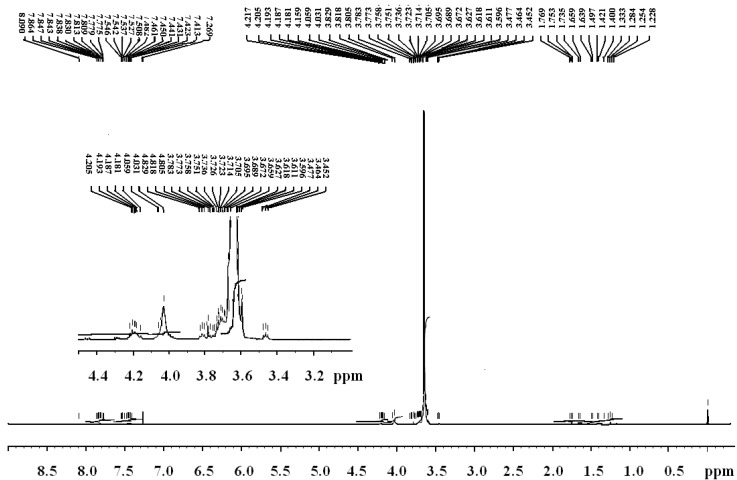
.^1^H-NMR spectrum of copolyphosphonate **4b**.

**Figure 3:**
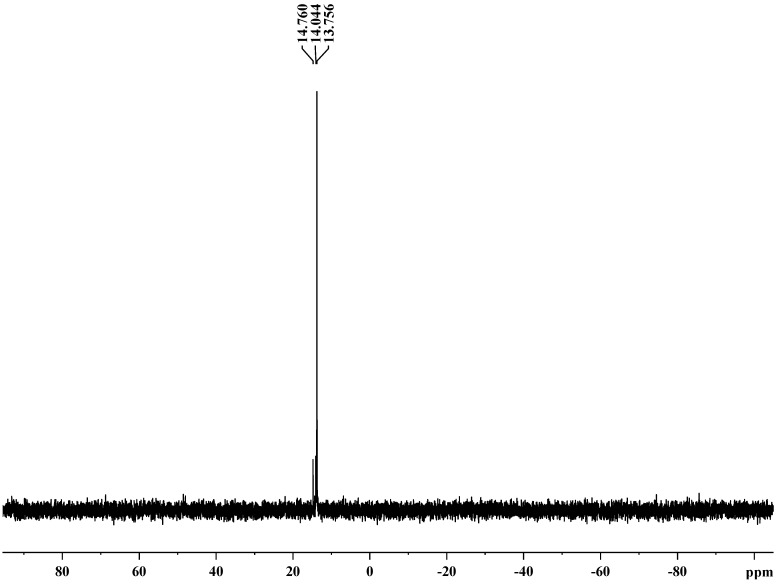
.^31^P-NMR spectrum of copolyphosphonate **4b**.

**Figure 4:**
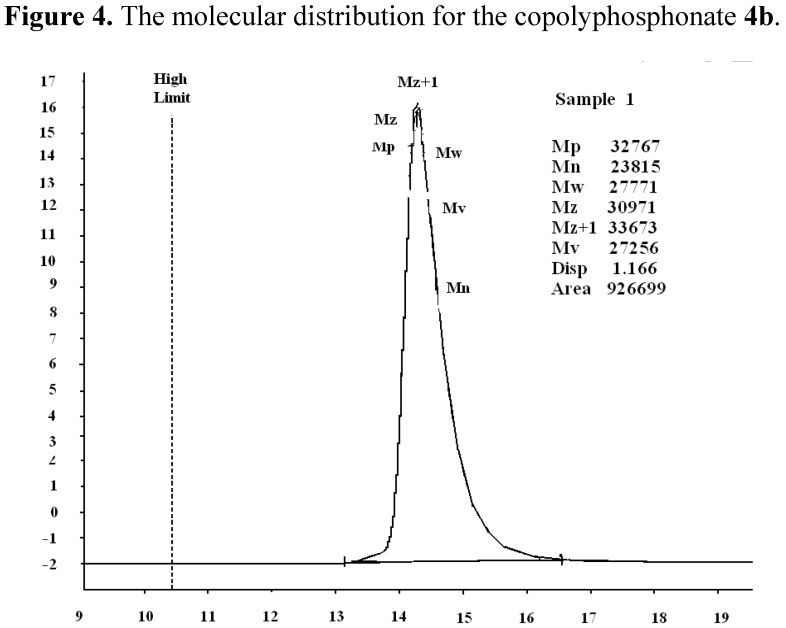
The molecular distribution for the copolyphosphonate **4b**.

**Figure 5:**
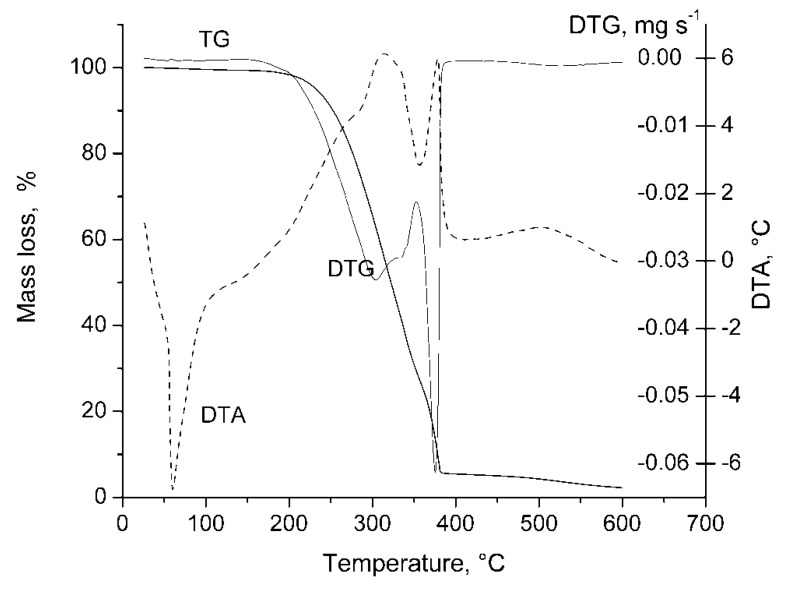
Thermoanalytical curves for polyphosphonate **4b**.

The chemical structures of (co)polyphosphonates **4a–c** were authenticated by %P, IR and NMR analysis. The spectral data were in according to the proposed structures.Representative spectra (IR, ^1^H and ^31^P-NMR spectra, respectively), molecular distribution and thermogravimetric data for the copolymer **4b** are shown in Figure 1, Figure 2, Figure 3, Figure 4, Figure 5.

The polymeric structure was supported by the P-O-C_aromatic_ and P-O-C_aliphatic_ str*etc*hing. The absorptions around 1240–1280 cm^−1^ correspond to ν_P=O_ which is characteristic of phosphonate ester compounds. Also, all the co(polymers) showed absorptions around 1180 and 960 cm^−1^ corresponding to P-C_arom_ str*etc*hing [28,29]. The resonance of the phenyl group falls in the range 6.2–7.9 ppm. Chemical shifts of the -CH_2_-OCH_2_- group were observed at the range of 3.4–3.7 ppm [30,31].

The presence of phosphorus is confirmed by elemental analysis and the ^31^P-NMR spectra. The %P content of these polymers is in agreement with the calculated values. The ^31^P-NMR spectra of polymers **4a** and **4c** present two signals: one corresponds to the P in the repeat unit and other one to the P at the chain end. The ^31^P-NMR spectrum of copolymer **4b** presents three signals: two correspond to the P in the repeat unit and other one to the P at the chain end. These data confirm successful incorporation of phosphorus in the polymer backbone [32,33].

The GPC measurements show the number average molecular weight is in the range of 5400–26300 (Table 1). The thermal stability of the (co)polymers **4a–c** was evaluated by the thermogravimetric analysis and flame retardancy by LOI (Table 2).

Char formation is important to flame retardancy because the carbonaceous char formed during degradation on the top of a polymer can protect the underlying polymer from exposure to the flame [34]. Char yield at 550 °C is higher in the case of **4c** and it is related to the phosphorus percent in units of mer. Also, the presence of phosphorus in **4b** increases the char content at 550 °C (2.3) compared with char content at 550 °C for pure PEG 12000 (0.8%) at the same temperature. This indicates an improvement of the flammability of copolymers. LOI measurements indicated their potential application as fire retardant materials being a precision method for determining the relative flammability of various materials by measuring the minimum concentration of oxygen required to support combustion. Limiting oxygen index (LOI) was determined for membranes according to ASTM D2863–1997 and for polymers on the powdered sample according to modified ASTM D2863–1997. These polymers show LOI values in the range 28–38, comparable with other polyphosphonates and polyphosphates [35].

**Table 2:** Characterization of polymers **4a–c**.

No	Weight loss correspondence to (°C)		T_m_, °C	Char yield at 550 °C, %	LOI
	5%	95%			
**4a**	222.2	378	55.12	1.8	28
**4b**	230.6	380	59.87	2.3	30
**4c**	190.4	420	-	2.6	38
**PEG**	320	420	74.63	0.8	23

The membranes based on **4a** and **4b**/LiCF_3_SO_3_ present a LOI value (25 and 27, respectively) close to those of **4a** and **4b**. The presence of the phosphonate group reduces flammability of the polymers and membranes.

In order to evaluate if the polymers **4a** and **b** can be used as solid polymer electrolytes, after the complexation with Li triflate, ionic conductivity and transference ion number were determined by means of impedance spectroscopy using stainless steel (SS) as blocking electrodes and the direct current (DC) method, respectively.

**Figure 6:**
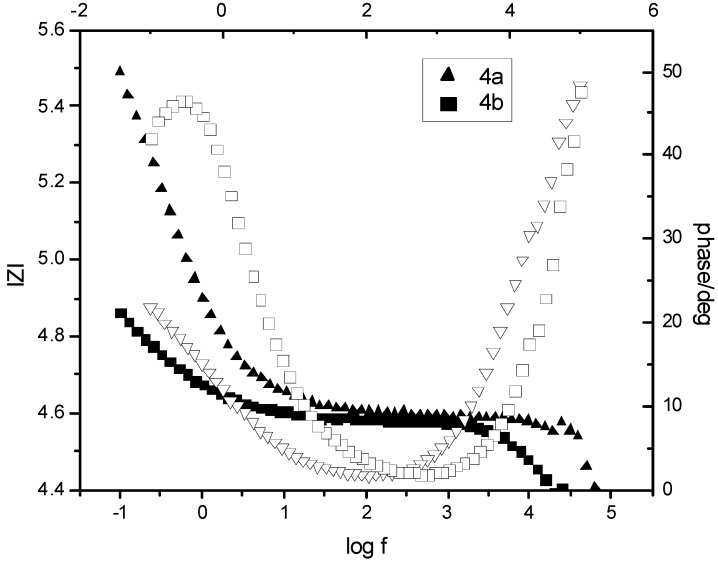
Bode plots at OCP potential, for the studied membranes sandwiched between two SS electrodes: 4a: **4a**/LiCF_3_SO_3_ and 4b: **4b**/LiCF_3_SO_3_.

**Figure 7:**
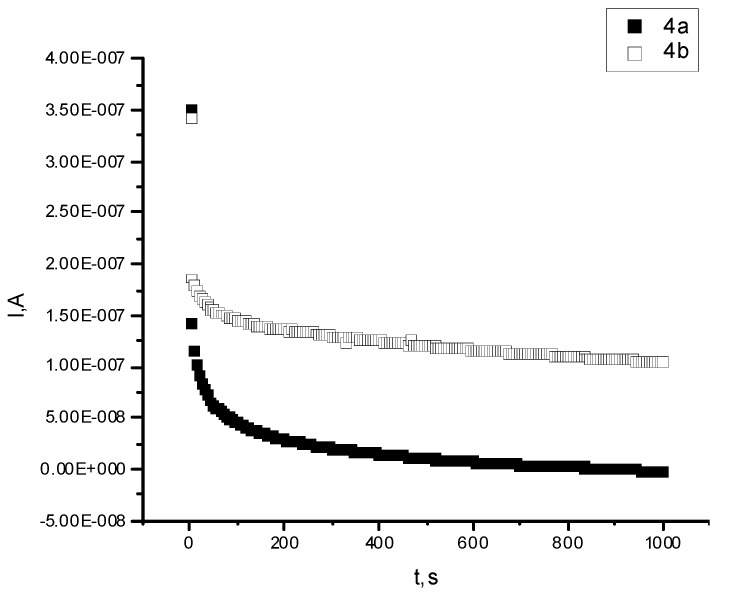
Polarization current as a function of time for membranes: 4a: **4a**/LiCF_3_SO_3_ and 4b: **4b**/LiCF_3_SO_3_.

The Bode plots for **4a**/LiCF_3_SO_3_ and **4b**/LiCF_3_SO_3_ at Open Circuit Potential (OCP), V, and the evolution of polarization current as a function of time after the application of a DC potential across the SS/**4a** and **b**/LiCF_3_SO_3_/SS cell are presented in Figure 6 and Figure 7.

The ionic conductivity (σ) value was calculated at room temperature according to Equation (1), from the intercept of the curve with real axis an the total ionic transference number was calculated from plots of the polarization current versus time with Equation (2). The conductivity for membrane **4a**/LiCF_3_SO_3_ was 1.93 × 10^−8^ S.cm^−1^ and for membrane **4b**/LiCF_3_SO_3_, was 6.37 × 10^−7^ S.cm^−1^, values which are higher than the conductivity reported in literature for pure PEG (about 1.67 × 10^–9^ S.cm^−1^) [36]. Also, in the case of membrane **4b**/LiCF_3_SO_3_ the conductivity is close to the value mentioned for the (PEG)*x*LiClO_4_ system [36]. Total ionic transference numbers calculated for membranes were 0.826 and 0.999 for **4a**/LiCF_3_SO_3_ and **4b**/LiCF_3_SO_3_, respectively. The values suggest that the charge transport in these polymer electrolyte membranes is predominantly due to ions.

## 3. Experimental

### 3.1. Materials

Reagents (PPD, PEG 12000, BA, and K_3_PO_4_) from Aldrich (Bucharest, Romania) were used as received and solvent (2-MeTHF- from Aldrich) was used without purification. Lithium trifluoromethanesulfonate (lithium triflate, LiCF_3_SO_3_) from Aldrich was used after drying under reduced pressure at 120 °C for 24 h. Methanol (without purification, Aldrich) and ethylene carbonate (EC, without purification, Aldrich) were used for membrane formation.

### 3.2. Procedure

#### 3.2.1. Synthesis of Phosphorus Containing (co)Polyesters by PTC Polycondensation in Solid-Liquid System [24]

A PTC polycondensation in solid-liquid system was run as shown in the following example for the synthesis of polymer **4a**: In a flask fitted with a stirrer, PEG 12000 (0.005 mol), K_3_PO_4_ (0.012 mol), and MeTHF (20 mL) were introduced. After stirring, a solution of PPD (0.005 mol) in MeTHF (10 mL) was added at once. During the addition, an exothermic reaction occurred. The mixture was still stirred for two hours at 40–45 °C. Following polycondensation, the salts were removed by filtration, and the polymer was purified by precipitation into an excess of hexane and isolated as a white solid which is dried under vacuum, at 50 °C and characterized. The yield was 86.5%.

For the synthesis of copolyester **4b** and polyphosphonate **4c** the same method was used, equimolecular amount of bisphenols (and/or 0.005 mol BA) being added (Scheme 2).

**4a**. IR (KBr, cm^−1^): 1239.04 (P=O), 1100.19; 841.77 (P-O-C_aliph_), 1462.74 (P-C_arom_); 2878.24(aliph-CH_2_-); 3048.3; 1343.18; 1620.91; 755.95 (Ph); ^1^H-NMR: 7.3–7.9 (m, C_6_H_5_); 3.72 (s, ‑CH_2_-O-CH_2_-); ^31^P-NMR: 14.7 (P at chain end), 13.7 (P in the repeat unit).

**4b**. IR (KBr, cm^−1^): 1240.97 (P=O); 1097.30; 840.812 (P-O-C_aliph_), 1463.71 (P-C_arom_), 2877.27 (aliph-CH_2_-), 3736.4; 1343.18; 1620.91; 755.95 (Ph); ^1^H-NMR: 7.30–7.90 (m, C_6_H_5_); 3.59–3.75 (m,-CH_2_-O-CH_2_-); 1.20–1.80 (m, C(CH_3_)_2_); ^31^P-NMR: 14.7 (P at chain end), 13.7 and 14.0 (P in the repeat unit).

**4c**. IR (KBr, cm^−1^): 1480 (P-C_arom_); 1280 (P=O); 940, 1200 (P(O)-O-C_arom_); ^1^H-NMR: 6.6–7.3 (m, C_6_H_5_); 1.2–1.6 (m, C(CH_3_)_2_); ^31^P-NMR: 15.7 (P at chain end), 12.0 (P in the repeat unit)

#### 3.2.2. Preparation of Phosphorus-Containing (co)Polyester Electrolytes

A certain amount of lithium salts (lithium triflate), around 10 wt %, was added to the (co)polymer **4a(**PPD- PEG**)** and **4b** (PPD-PEG-BA/) solutions (methanol was used as a solvent) and stirred until the lithium salt is dissolved. Ethylene carbonate (2%) was added as a plasticizer to a phosphonate–polyether network to improve the ionic conductivity at room temperature. Then the mixture was cast on a Teflon plate and dried under vacuum at 70 °C for 24 h to form polymer electrolyte complex films. The membrane notation is **4a**/LiCF_3_SO_3_ and **4b**/LiCF_3_SO_3_.

### 3.3. Analysis

The IR spectra were recorded on a JASCO-FT/IR-4200 spectrophotometer and ^1^H-NMR and ^31^P-NMR spectra on a Bruker DRX 400 MHz spectrometer. All NMR spectra were recorded in CDCl_3 _using TMS as internal standard, at 25 °C. The polymers were characterized by viscosity, on an Ubbelohde suspended level viscometer, at 30 °C and by gel permeation chromatography (GPC), on an Evaporative Light Scattering Detector, PL-EMD 950 (2 × PL gel MIXEDC 300 × 7.5 mm columns; T = 35 °C; DMF as solvent; Flow 1 mL/min; calibration with KIT polystyrene as standard). The thermoanalytical curves TG, DTG and DTA (as heat flow) were drawn up by a TGA/SDTA 851-LF 1100-Mettler Toledo device, in a nitrogen atmosphere and heating rates of 10 deg∙min^−1^. Limiting oxygen (LOI) index was determined using a Limiting Oxygen Index Chamber 340AJH0038 according to the ASTM D2863–1997 standard for membranes. For polymers the method was modified as follows: about 1 g of polymer was placed in a glass cup. An external flame of 20 mm length was maintained in contact for 10 s with the polymer [35].

Ionic conductivity of the **4a** and **4b** films was determined by AC impedance spectroscopy. The impedance tests were carried out in the frequency range from 0.1 Hz to 10^6^ Hz using an Autolab 302N potentiostat/galvanostat equipped with the FRA2 impedance module. The sinusoidal potential amplitude was 10 mV. All electrochemical measurements were performed at room temperature (ambient condition). For each spectrum 60 points were collected, with a logarithmic distribution of 10 points per decade. The sample films were sandwiched between symmetrical cells containing blocking stainless steel (SS) electrodes. Analysis of the impedance spectra is based on the Bode diagrams. At the point where the phase angle is zero (or close to zero), the impedance is pure ohmic and the resistance of the membrane can directly be determined and used for the ionic conductivity calculation, by the following equation (1):

σ = L/Rb.A (1)

where:

σ—ionic conductivity,

Rb—the resistance corresponding to the angle closest to zero in the Bode diagram,

L—the height of the sample between the electrodes,

A—the cross-sectional contact area of the measured sample with the electrodes.

Transference numbers were evaluated with Wagner’s polarization technique [29]. The total ionic transference number was calculated from plots of the polarization current *versus* time with the equation (2):

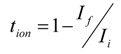
(2)
where *I_i_* is the initial current and *I_f_* is the final residual current.

## 4. Conclusions

The aim of this work was to apply the phase-transfer catalysis method, in a solid-liquid system, for the first time in the synthesis of phosphorus solid polymer electrolytes, as an eco-friendly and economical procedure for the synthesis of phosphorus-containing polymers, using potassium phosphate.

Phosphorus-based electrolytes that are safe and non-flammable were synthesized. This paper presents the synthesis by solid-liquid phase transfer catalysis of linear solid (co) polyphosphonates by polycondensation of phenylphosphonic dichloride with poly(ethylene glycol) 12,000 with and without bisphenol A. Potassium phosphate is used as base.

The novelty of this study lies in the synthesis of new linear solid (co) polyphosphonates based on PPD, PEG and BA (**4b**). The properties of the (co)polymers such as thermal stability and flammability have been investigated. Ionic conductivity of (co)polyphosphonates **4a,b** was investigated too.

These (co)polymers showed good LOI values (in the range 28–38). The membranes based on **4a** and **4b**/LiCF_3_SO_3_ present a LOI value (25, respectively 27) close to the **4a** and **4b** and indicates an improvement of the safety of lithium batteries. The presence of the phosphonate group reduces the flammability of the polymers and membrane.

The **4b**/LiCF_3_SO_3_ electrolyte exhibited ionic conductivity of 6.37 × 10^−7^ S·cm^−1^ at 25 °C, higher than **4a**/LiCF_3_SO_3_. Also, the total transference number closely to unitary of this membrane indicates that this copolyphosphonate can be considered as a candidate for solid polymer electrolytes. The charge transport in Li triflate-polyphosphonate electrolyte membrane is predominantly due to ions. 
